# Investigation of Energy Transfer in Star-Shaped White Polymer Light-Emitting Devices via the Time-Resolved Photoluminescence

**DOI:** 10.3390/ma11091719

**Published:** 2018-09-14

**Authors:** Hui He, Xiaoqing Liao, Jiang Cheng, Ying Li, Junsheng Yu, Lu Li

**Affiliations:** 1 State Key Laboratory of Electronic Thin Films and Integrated Devices, School of Optoelectronic Science and Engineering, University of Electronic Science and Technology of China (UESTC), Chengdu 610054, China; hhe@std.uestc.edu.cn (H.H.); jsyu@uestc.edu.cn (J.Y.); 2 Co-innovation Center for Micro/Nano Optoelectronic Materials and Devices, Research Institute for New Materials and Technology, Chongqing University of Arts and Science, Chongqing 402160, China; xiaoqin5122@163.com (X.L.); jiangcheng@cqwu.edu.cn (J.C.); leoyingchem@163.com (Y.L.)

**Keywords:** star-shaped white polymer light-emitting devices, energy transfer, time-resolved photoluminescence, efficiency roll-off

## Abstract

A series of white polymer light-emitting devices (WPLEDs) were fabricated by utilizing star-shaped white-emission copolymers containing tri[1-phenylisoquinolinato-C2,N]iridium (Ir(piq)_3_), fluorenone (FO) and poly(9,9-dioctylfluorene) (PFO) as red-, green- and blue-emitting (RGB) components, respectively. In these WPLEDs, a maximum current efficiency of 6.4 cd·A^−1^ at 20 mA·cm^−2^ and Commission Internationale d’Eclairage (CIE) coordinates of (0.33, 0.32) were achieved, and the current efficiency was still kept to 4.2 cd·A^−1^ at the current density of 200 mA·cm^−2^. To investigate energy transfer processes among the three different chromophores of the star-shaped copolymers in these WPLEDs, the time-resolved photoluminescence (PL) spectra were recorded. By comparing the fluorescence decay lifetimes of PFO chromophores in the four star-like white-emitting copolymers, the efficient energy transfer from PFO units to Ir(piq)_3_ and FO chromophores was confirmed. From time-resolved PL and the analysis of energy transfer process, the results as follows were proved. Owing to the star-like molecular structure and steric hindrance effect, intermolecular interactions and concentrations quenching in the electroluminescence (EL) process could also be sufficiently suppressed. The efficient energy transfer also reduced intermolecular interactions’ contribution to the enhanced device performances compared to the linear single-polymer white-light systems. Moreover, saturated stable white emission results from the joint of energy transfer and trap-assisted recombination. This improved performance is expected to provide the star-like white-emitting copolymers with promising applications for WPLEDs.

## 1. Introduction

In the last few decades, white polymer light-emitting devices (WPLEDs) have gained remarkable attention because of their various applications in full-color displays with color filters, backlights, and solid-state lighting [1,2,3,4,5,6,7,8]. The low-cost solution processing offers WPLEDs great advantages over white organic light-emitting devices (WOLEDs) based on small molecules [9,10,11]. To fabricate WPLEDs with high performance, multilayer systems and blend systems have been widely employed; however, owing to the mixing of adjacent layers and phase separation, the device performances are not satisfactory. In contrast, the single-polymer white-emission systems can accomplish comparably stable bias-independent electroluminescence (EL) spectra, therefore, different kinds of single-polymer white-emission systems including linear white-emitting polymer systems [12,13,14] and star-shaped white-light polymer systems [15,16] have already been developed. Compared with linear white-light polymers, due to effectively suppressed intermolecular interaction in the emitting layer (EML), the star-shaped white-light polymers exhibit higher EL efficiency for WPLEDs and have been more widely studied [17,18]. Wang et al. [19] recently reported a star-shaped white electroluminescent single-polymer system that employed two kinds of fluorescent dyes as orange cores and blue arms, respectively. A high current efficiency of 18 cd·A^−1^ was obtained in their single-layer devices utilizing the star-like white-emitting polymers as the active layer. Yang and his coworkers [20] also developed a series of new two-color star-shaped white-emission single polymers that simultaneously consist of fluorescent and phosphorescent dyes. However, as these star-shaped white-emitting polymers only comprise two complementary colors, the EL spectra of these polymers cannot cover the whole visible range and result in the limitation of applications.

In order to realize the saturated three-color white light, great efforts have been made by several groups to develop three-color star-shaped polymers with high-color-quality saturated white EL [21,22]. At the same time, in order to fabricate more efficient WPLEDs and further enhance EL performances of white-emitting polymers, many groups have also studied the mechanisms of white emission within the star-shaped white polymers [23,24,25]. But these previous investigations only involved two-color star-like copolymers or all-fluorescent star-like copolymers. Moreover, the energy transfer in three-color star-shaped white-emitting copolymers which comprise both fluorescent and phosphorescent chromophores has not yet been fully disclosed. Additionally, as an effective method that clarifies energy transfer in light-emitting devices, the time-resolved photoluminescence (PL) has been widely employed among blend light-emitting systems [26,27,28]; however, the investigation of energy transfer via the time-resolved photoluminescence on the star-like light-emitting copolymers is still scarce.

Therefore, the current work was carried out to investigate energy transfer between the host chromophores and guest chromophores in the three-color star-shaped white-light copolymers. In this work, a series of star-shaped copolymers reported previously [29], which comprise Ir(piq)_3_ core and poly(9,9-dioctylfluorene) (PFO) arms endcapped with a fluorenone (FO) unit, were utilized to fabricate the WPLEDs. The time-resolved PL of star-shaped copolymers both in dilute solution and in neat films were employed to demonstrate energy transfer processes in these three-color star-shaped copolymers. In addition, the energy transfer was classified by the PL spectra and EL spectra.

## 2. Materials and Methods

### 2.1. Materials

**Figure 1:**
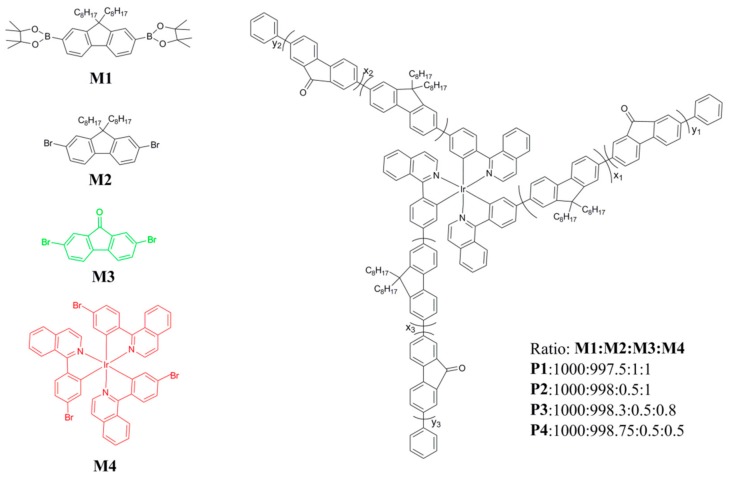
The chemical structures of the monomers and star-shaped white-emission copolymers.

In our work, the star-like white copolymers were synthesized according to the reported synthesis procedure. The chemical structures of the monomers and the star-shaped copolymers are depicted in Figure 1. In the star-shaped copolymers, Ir(piq)_3_, FO and PFO (Energy Chemical, Shanghai, China) act as the red-, green- and blue-light-emitting units, respectively. The four kinds of monomers (M1, M2, M3 and M4) were synthesized into the star-shaped copolymers P1, P2, P3 and P4 at the different feed ratios of 1000:997.5:1:1, 1000:997.8:0.5:1, 1000:998.3:0.5:0.8 and 1000:998.75:0.5:0.5, respectively. Detailed synthesis procedures are described in [29].

### 2.2. The Fabrication of Devices and Measurements

**Figure 2:**
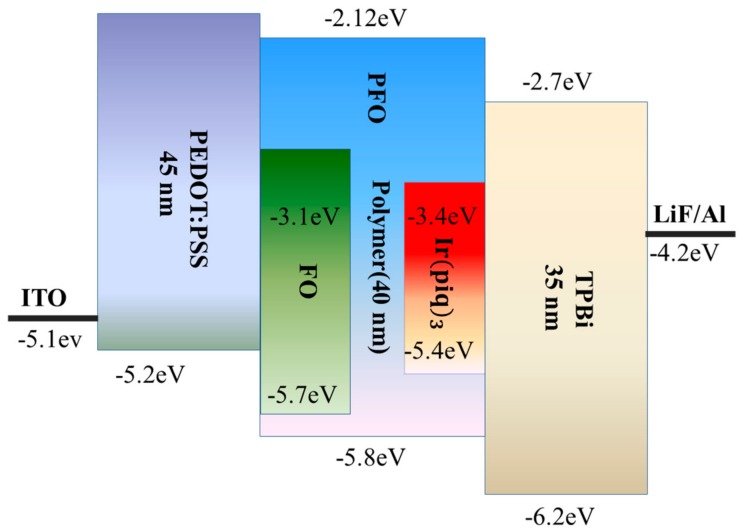
Device structure and energy levels of the materials.

WPLEDs were fabricated on the indium tin oxide (ITO)/glass substrates with a sheet resistance of ~15 Ω/sq. First of all, the ITO/glass substrates were cleaned successively with detergent, deionized water, acetone and isopropanol for 15 min each step under ultrasonication, and then the substrates were treated with oxygen plasma to increase the work function and decrease the surface roughness. Poly(3,4-ethylenedioxythiopene):poly(styrenesulfonate) (PEDOT:PSS) (Heraeus, Shanghai, China) was spin-coated on the ITO/glass substrates at 3000 rpm for 60 s and then baked at 130 °C. The resulting thin films were approximately 45 nm. The emitting materials in chlorobenzene (10 mg·mL^−1^) (Sigma, Shanghai, China) were then spin-coated onto the PEDOT:PSS layer, and the solvent residue was removed after the emissive layer was annealed at 100 °C for 30 min, and the resulting film was approximately 40 nm. Finally, a layer of 1,3,5-Tri(1-phenyl-1H-benzo[d]imidazole-2-yl)phenyl (TPBi (35 nm)) (Xi’an Polymer Light Technology Corp, Xi’an, China ), a thin layer of LiF (1 nm) and a layer of aluminum (100 nm) were successively deposited in a vacuum thermal evaporator through a shadow mask at a base pressure of 5 × 10^−4^ Pa. The resulting WPLEDs (devices A1, A2, A3, and A4, utilizing the polymers P1, P2, P3 and P4 as emitters, respectively) had a sandwiched configuration. Device structure and energy levels of the materials are shown as Figure 2. The active area of the devices was 0.12 cm^2^. Luminance–current density–voltage (L–J–V) characteristics were measured with a Keithley 2400 source (Keithley, Shanghai, China) and a calibrated silicon photodetector (Keithley, Shanghai, China). The EL spectra, Commission Internationale d’Eclairage (CIE) coordinate and the color rendering index (CRI) were recorded using a photoresearch PR-670 (Photo Research, Shanghai, China). The device fabrication and testing were carried out in a nitrogen-filled dry box with oxygen and moisture levels both below 0.1 ppm.

## 3. Results and Discussions

### 3.1. Electrical Characteristics of the Devices

**Figure 3:**
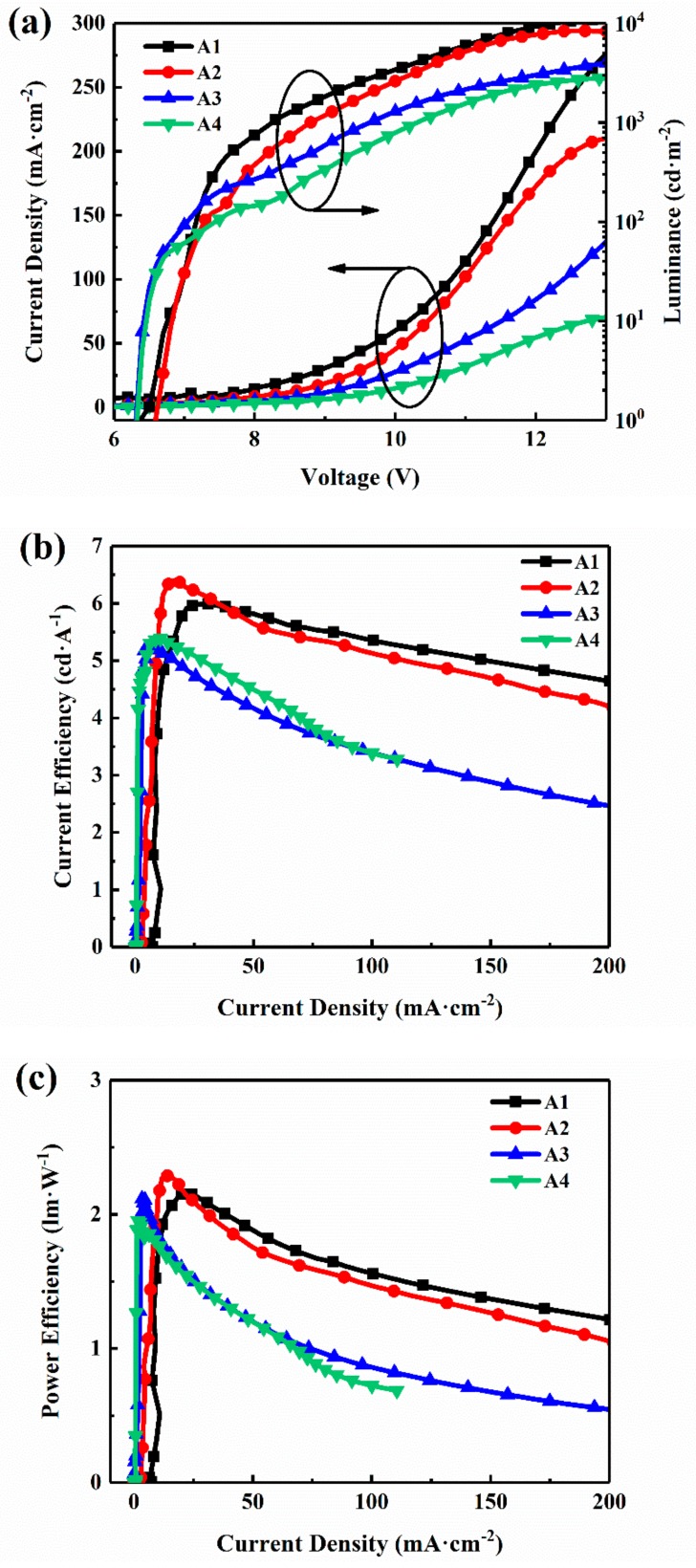
The performances of devices. (**a**) The current density–luminance–voltage (J–L–V) characteristics, (**b**) the current efficiency–current density (CE–J) characteristics, (**c**) the power efficiency–current density (PE–J) characteristics.

Figure 3a shows the L–J–V characteristics of the devices (see the double-logarithmic JV curves in Figure S1). It could be learned from the L–V curves that all devices show relatively high turn-on voltages (voltage at a luminance of 1 cd/m^2^), ranging from 6.2 V to 6.5 V, and the luminance of the devices decreases with reducing content of FO and Ir(piq)_3_, and the maximum luminance of the A1, A2, A3 and A4 devices can reach 10,960 cd·m^−2^, 8421 cd·m^−2^, 5433 cd·m^−2^ and 3627 cd·m^−2^, respectively. Meanwhile, the J–V curves demonstrate a similar tendency with luminance of the devices from device A1 to device A4. Reasonable explanations for the current density and luminance behaviors of the devices can be given as follows. It can be seen from Figure 2 that the lowest unoccupied molecular orbital (LUMO) and highest occupied molecular orbital (HOMO) energy levels of FO (estimated to be −3.1 eV and −5.1 eV, respectively) and Ir(piq)_3_ (estimated to be −3.4 eV and −5.4 eV, respectively) lie between the LUMO (−2.12 eV) and HOMO (−5.8 eV) energy levels of PFO. When electrons and holes are injected into EML from the layers of PEDOT:PSS and TPBi, respectively, the sites of FO and Ir(piq)_3_ will act as traps for both electrons and holes in EML [26,30,31] and these carriers are trapped by these traps near the interface of EML, hindering the migration of these carriers towards the recombination region [32,33]. Therefore, more carriers need to be injected into the EML for white emission, which causes a larger current density. With increasing content of the red and green units (from device A4 to device A1), more triplet energy is utilized to produce light emission in the recombination region and lead to the higher device luminance. Figure 3b,c plot the current efficiency–current density (LE–J) characteristics and power efficiency–current density (PE–J) characteristics of WPLEDs, respectively. It can be observed that, with the content of Ir(piq)_3_ and FO increasing, the device efficiencies gradually enhance. The maximum current efficiencies and power efficiencies for the device A1–A4 are 5.9 cd·A^−1^ and 2.1 lm·W^−1^, 6.4 cd·A^−1^ and 2.3 lm·W^−1^, 5.2 cd·A^−1^ and 2 lm·W^−1^, 5.3 cd·A^−1^ and 1.8 lm·W^−1^, respectively. Figure 3b shows that the current efficiencies of device A1 and A2 still maintain high values at a current density 200 mA·cm^−2^, and the low efficiency roll-off is quite suitable for applications in flexible displays. The performances of WPLEDs are summarized in Table 1, and we can see that the device A2 exhibits the best performances with a high rendering index (CRI) of 91, and the 1931 CIE coordinates are (0.33, 0.32) (Figure S2), which are very close to standard white emission, (0.33, 0.33).

**Figure 4:**
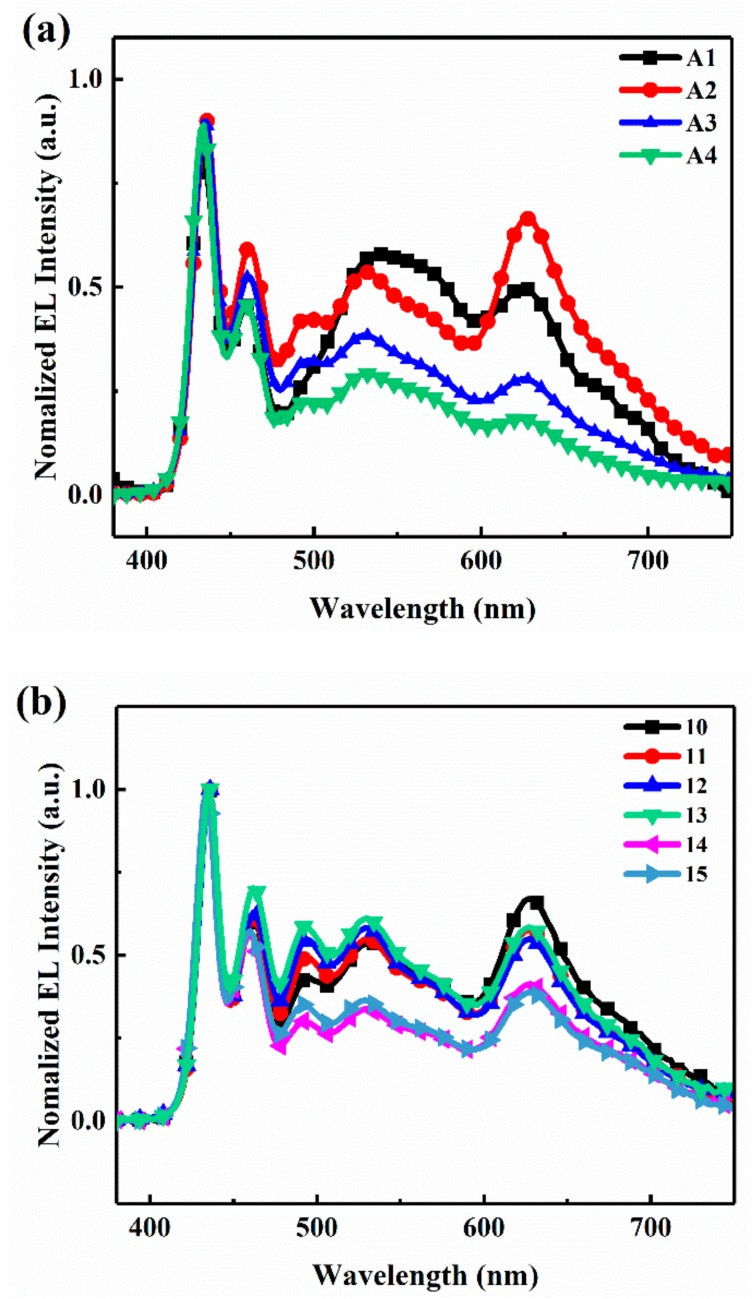
The spectral behaviors. (**a**) The normalized electroluminescence (EL) spectra of device A1–A4 at a driving voltage of 10 V, (**b**) the normalized EL spectra of device A2 at different voltages.

Figure 4a shows the normalized EL spectra of WPLEDs and the blue emission peaks (432 nm and 460 nm), green emission peak (540 nm) and red emission peak (628 nm) originating from PFO, FO and Ir(piq)_3_ chromophores, respectively. It can be seen that with the increase of the dopant units content from copolymers P4-P1, the relative intensity of red emission and green emission peaks gets stronger (from the device A4 to A1). Figure 4b shows the EL spectra of device A2 at different voltages (10–15 V); the spectral shapes show slight changes in the range of 10 to 13 V, and the stable saturated white light is suitable for panel display and lighting. However, the charges become saturated for traps as the voltages are increased further, and more carriers generate radiative recombination on FPO arms and produce strong blue emission.

### 3.2. The Time-Resolved Photoluminescence and Energy Transfer within the White-Light Copolymers

**Figure 5:**
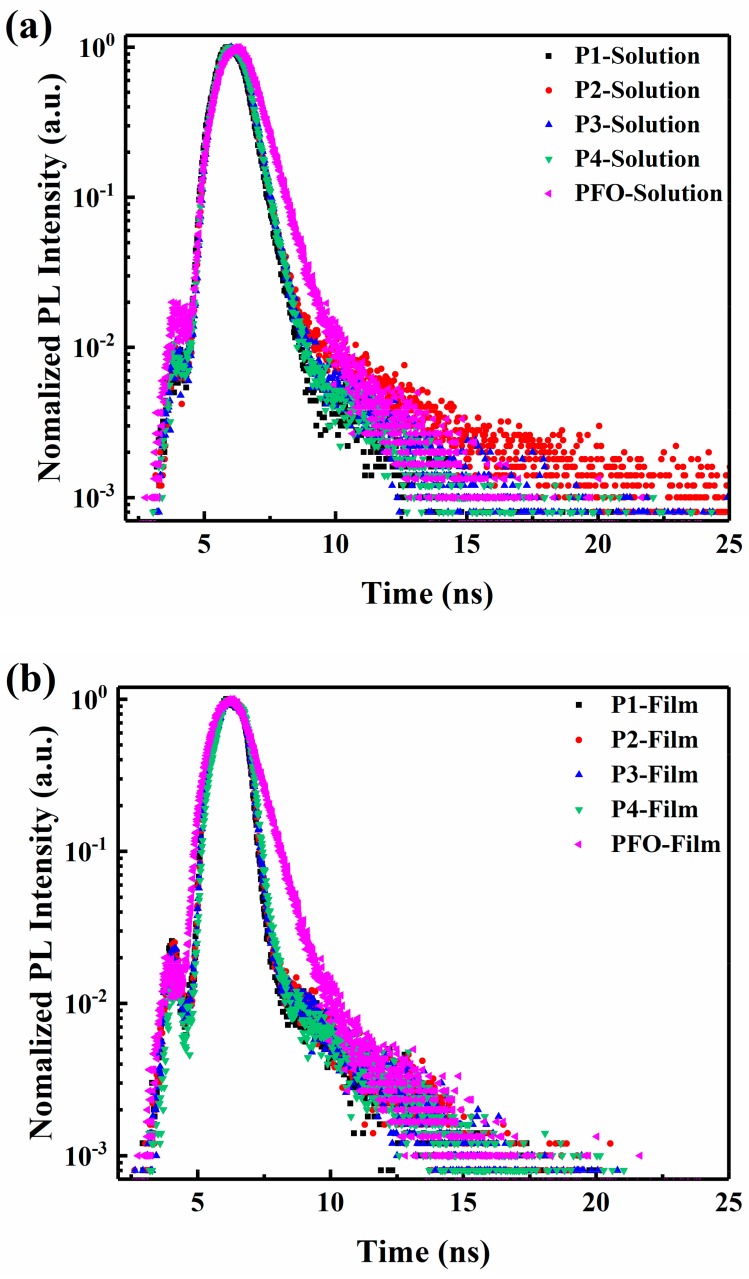
The time-resolved photoluminescence (PL) transients of P1–P4 and poly(9,9-dioctylfluorene) (PFO), (**a**) in Tetrahydrofuran(THF) solution (5 × 10^−6^ M), (**b**) in neat films (λ_ex_ = 375 nm, λ_em_ = 423 nm).

In order to clarify the efficient energy transfer from PFO moieties to FO and Ir(piq)_3_ units in EL process, the time-resolved fluorescence decays for PFO segments of the copolymers P1–P4 and the pure PFO (linear PFO) both in solutions and in films were recorded as plotted in Figure 5a,b, respectively. The fluorescence decay can be well-fitted with a monoexponential decay curve. The fitting curve (Figure S3) can be described with Equation (1),

(1) $$I_{\mathrm{PL}}\left(t\right)=I_{0}\exp[-(t/\tau^{*})]+C$$

I_PL_ is the normalized photoluminescence intensity, and the constants I_0_ and C represent the relative initial intensity of the emitted light at the end of the laser pulse and the relative background intensity due to the electrical driving and noise, respectively. The parameter τ* represents the PL lifetime and is the main parameter in the fitting process [34]. The corresponding fluorescence lifetime values are listed in the Table 2.

As shown in Figure 5a, the fluorescence transient decays in copolymer solutions are shorter than that in pure PFO solution; this is because for excited energy of pure PFO there are only two decay channels: the radiative decay channel and non-radiative relaxation, but for the excited energy of PFO in copolymers, there is the third decay channel: energy transfer, hence the excited PFO of copolymers have a faster relaxation and a shorter lifetime than that of pure PFO. It can be also seen from Table 2 that the fitted fluorescence lifetimes of copolymer P1–P4 in solution are 467 ps, 471 ps, 472 ps and 470 ps, respectively, and there is no clear difference to these lifetimes for the white emitting polymers P1–P4, which demonstrates relatively weak intramolecular energy transfer. The fitted decay lifetimes of PFO segments for copolymer P1, P2, P3 and P4 neat films are 221 ps, 230 ps, 240 ps and 263 ps, respectively, and are shorter than that in solution, which verifies more efficient energy transfer from PFO chains to Ir(piq)_3_ and FO chromophores in copolymer neat films. The detailed energy transfer processes among the white-light polymers solution and neat films can be explained as follows. First of all, in ultra-dilute white-light polymer solutions, the white-emitting polymers are regarded as independent and separate from each other, and the intermolecular distance is beyond the $F\ddot{o}\mathrm{rster}$ resonant energy transfer (FRET) radius R_0_, so intermolecular energy transfer from PFO chains to Ir(piq)_3_ and FO units of adjacent white-emitting polymers are negligible. But in the same copolymer, singlet excitons produced in the fluorenes which are close to Ir(piq)_3_ and FO chromophores could migrate to these red- and green-emission chromophores through intramolecular FRET processes. With the increase of conjugation and aggregation of copolymers in neat films, the distance of donor-accepter separation would typically be smaller than the $F\ddot{o}\mathrm{rster}$ radius R_0_; hence, intermolecular energy transfer from PFO segments to Ir(piq)_3_ and FO segments are enhanced greatly. The combination of intramolecular and intermolecular transfer processes leads to a smaller fluorescence lifetime τ for the copolymer neat films. The fluorescence transient decays of the white-emission polymers both in solutions and in films present a long trail, and this phenomenon can be attributed to the existence of exciplexes, which lead to delayed emission.

On the other hand, the fluorescence lifetime corresponding to the blue emission of star-like PFO is around 520 ps [25], which is longer than that of linear PFO (380 ps) in the current work, indicating that the star-shaped structure can sufficiently suppress intermolecular interaction and reduce non-radiative relaxation. Additionally, the fluorescence decays get slower from P1 to P4, which demonstrates that energy transfer gradually becomes weakened, which is in accordance with the lower content of Ir(piq)_3_ and FO chromophores leading to larger donor–accepter separation.

**Figure 6:**
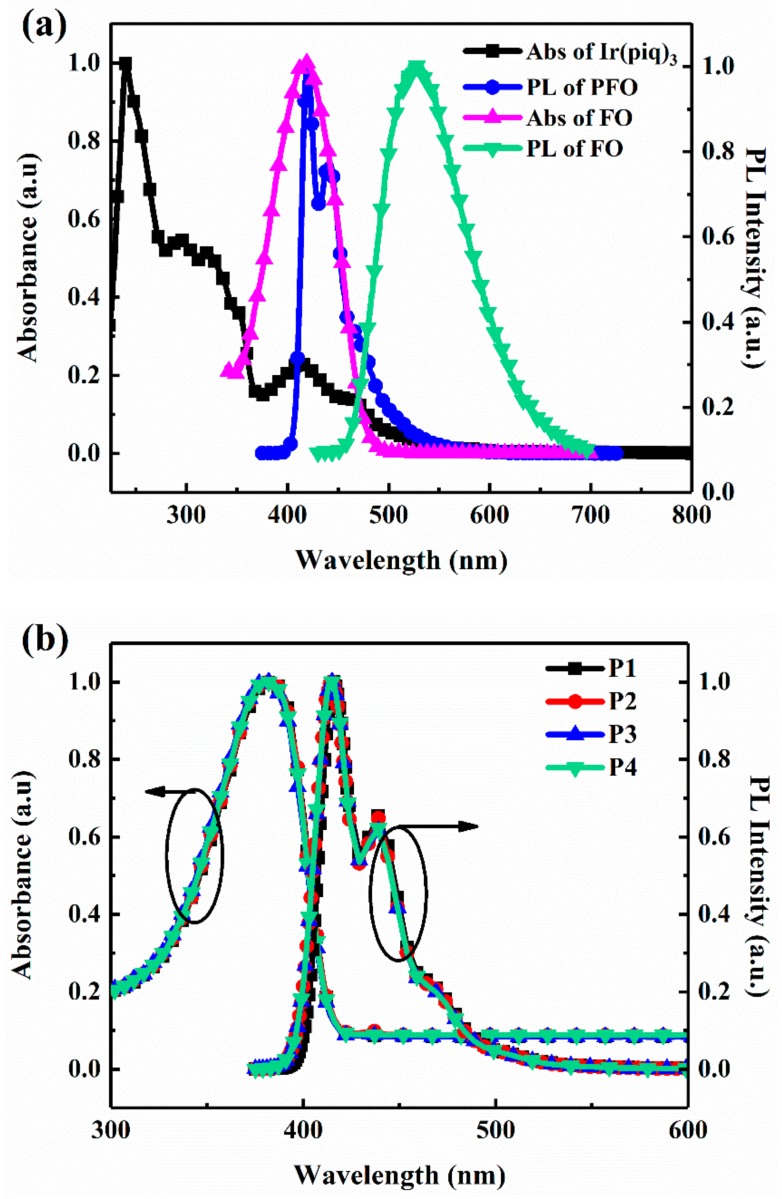

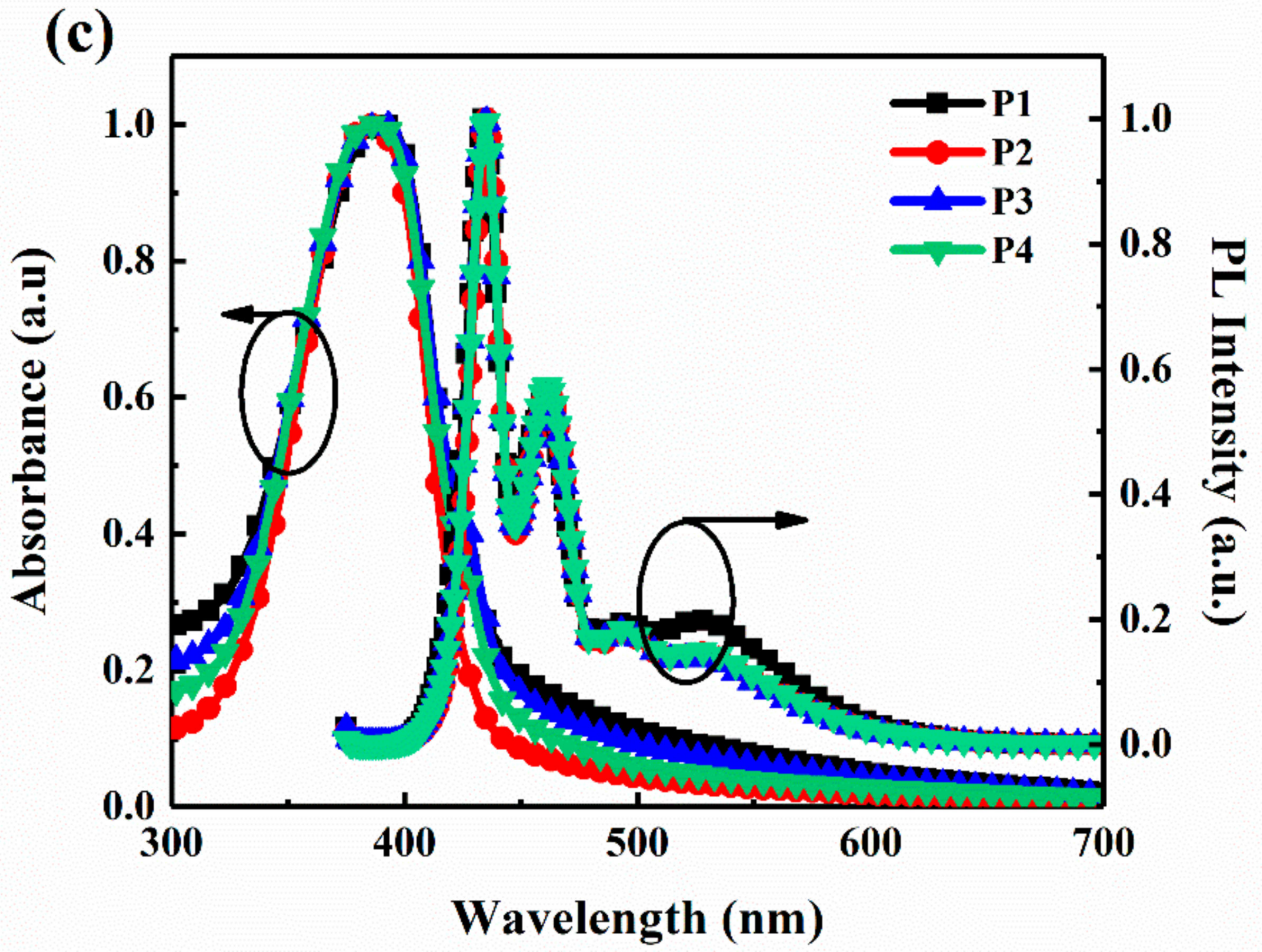
The ultraviolet–visible (UV–vis) absorption spectra and the normalized PL spectra, (**a**) the UV–vis absorption spectra of Ir(piq)_3_ and fluorenone (FO) and the PL spectra of PFO and FO in THF solutions (5 × 10^−6^ M), and (**b**) the UV–vis absorption spectra and the normalized PL spectra of copolymers P1–P4 in THF solutions (5 × 10^−6^ M), (**c**) the UV–vis absorption spectra and the normalized PL spectra of copolymers P1–P4 in neat films (λ_ex_ = 375 nm, λ_em_ = 423 nm).

The different energy transfer mechanisms are also certified via the ultraviolet–visible (UV–vis) absorption spectra and the PL spectra of the monomers and the white-light copolymers. As shown in Figure 6a, there is an overlapped band between the UV–vis absorption spectrum of Ir(piq)_3_ and the PL spectrum of PFO, indicating FRET from PFO segments to Ir(piq)_3_ phosphor. Compared with Ir(piq)_3_, there is a larger overlapped band between the UV–vis absorption spectrum of FO and the PL spectrum of PFO, indicating more efficient FRET from PFO segments to FO. The UV–vis absorption spectra and the PL spectra of the light-emitting copolymers P1–P4 in solutions and in films are displayed in Figure 6b,c, respectively. All copolymers both in solutions and in films present a very similar UV–vis absorption band with a peak wavelength at about 380 nm, which is assigned to the π–π* transition of PFO backbone. It can be also seen in Figure 6b that the main emission peaks in solution are located at about 420 nm and 440 nm, and the emission peak at 440 nm comes from the exciplex emission, which is consistent with a long trail of the fluorescence transient decays. Moreover, because the intramolecular energy transfer is so weak that no red and green emission are visible [13]. However, the PL spectra in films showed in Figure 6c exhibits the main blue emission peaks as well as the weak green emission originating from FO moieties, which is because simultaneous intramolecular and intermolecular FRET processes generate more excited FO units and result in stronger green emission peaks. The energy transfer efficiency (E_t_) could be calculated from Equation (2) [35,36], where $\tau_{0}$ is the lifetime of star-like PFO and  τ ′ is the lifetime of copolymer films. The E_t_ is also listed in Table 2.

(2) $$E_{t}=1-\frac{\;\tau \;\prime }{\tau_{0}}$$

It can be found that the values of transfer efficiency are gradually reduced from copolymer P1 to copolymer P4 with decreasing dopant unit content.

**Figure 7:**
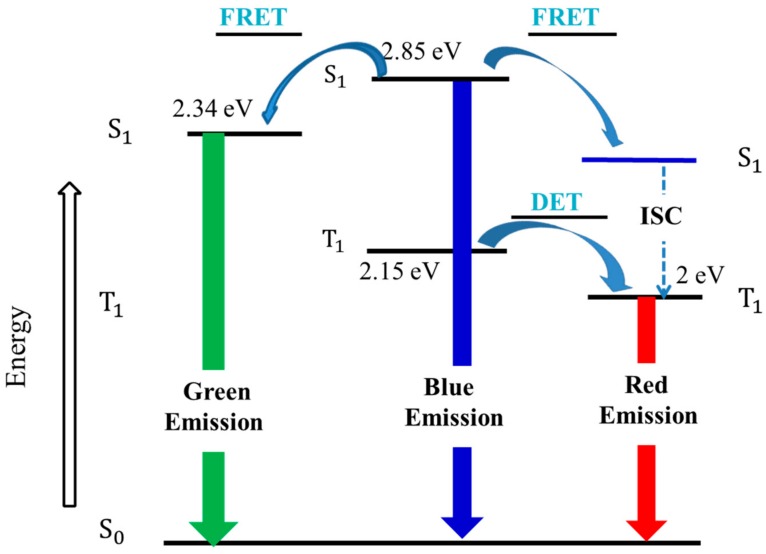
Exciton energy diagram of PFO, FO and Ir(piq)_3_ and energy transfer processes.

Additionally, due to more trap-assisted radiative recombination and more triplet emission in EL processes, the EL spectra show stronger green emission peaks and red emission peaks than those of PL spectra [37]. Figure 7 indicates the energy transfer processes among the different chromophores of the white-emission polymers in the EL process. The combination of efficient energy transfer and trap-assisted recombination generates saturated white emission, as is referred to from CE–J characteristics above, and the reduced intermolecular interaction resulting from the special spatial structure and energy transfer from PFO moieties to FO and Ir(piq)_3_ units contribute to low efficiency roll-off [38,39].

## 4. Conclusions

In conclusion, a series of star-shaped WPLEDs were fabricated. Compared to the star-shaped WPLEDs reported previously, low-efficiency roll-off and saturated white emission in our systems were obtained. The comparison between the time-resolved fluorescent spectra shows that the excitation energy of PFO chains can efficiently transfer onto the FO and Ir(piq)_3_ units in neat films by the FRET process containing intramolecular and intermolecular processes, and the star-shaped molecular structure can efficiently reduce intermolecular interaction. Through the analyses of PL spectra and EL spectra, we found that the white emission is due to the double effects of energy transfer and trap-assisted recombination in EL processes. Based on these conclusions, the EL performances of the promising star-shaped white-emitting copolymers containing both fluorescent and phosphorescent chromophores can be further enhanced by optimizing the device structure of WPLEDs.

## Figures and Tables

**Table 1:** Performance for white polymer light-emitting devices (WPLEDs) with polymers P1, P2, P3 and P4 as white-light emitters, respectively.

Device	Composition (Green:Red)	Max CE (cd·A^−1^)	Max PE (lm·W^−1^)	CIE *^a^*	CRI *^a^*
A1	1:1	5.9	2.1	(0.34, 0.35)	83
A2	0.5:1	6.4	2.3	(0.33, 0.32)	91
A3	0.5:0.8	5.2	2	(0.27, 0.34)	86
A4	0.5:0.5	5.3	1.8	(0.27, 0.26)	65

***^a^*** Commission Internationale d’Eclairage (CIE) and rendering index (CRI) were measured at a driving voltage of 10 V.

**Table 2:** The quantum yields, PL decay lifetime and energy transfer efficiency of polymers.

Polymer	$\Phi_{\mathrm{PL}}(\%)$		τ(ps)		E_t_ (%)
	Solution	Film	Solution	Film	
PFO	85	40	676	380	–
P1	80	28	467	221	57.5
P2	83	47	471	230	55.7
P3	84	42	472	240	53.8
P4	88	39	470	263	49.4

## Supplementary Materials

The following are available online at http://www.mdpi.com/1996-1944/11/9/1719/s1, Figure S1: The double-logarithmic JV curves, Figure S2: The 1931 CIE chromaticity diagram of emission of the WPLEDs; (**a**) the CIE coordinates of devices A1–A4 at a voltage of 10 V; (**b**) the CIE chromaticity diagram of device A2 at different voltages, Figure S3: The fit curves of the time-resolved PL for copolymers P1-P4; (**a**) the white-light copolymer in solutions; (**b**) the white-light copolymer in films.
