# Supplementary material for: Investigation of Energy Transfer in Star-Shaped White Polymer Light-Emitting Devices via the Time-Resolved Photoluminescence
Source: Materials (Basel). 2018 Sep 14;11(9):1719. doi: 10.3390/ma11091719 (PMC6163793; doi:10.3390/ma11091719)
Supplement: Supplementary file 1 [file materials-11-01719-s001.pdf]

Supporting information

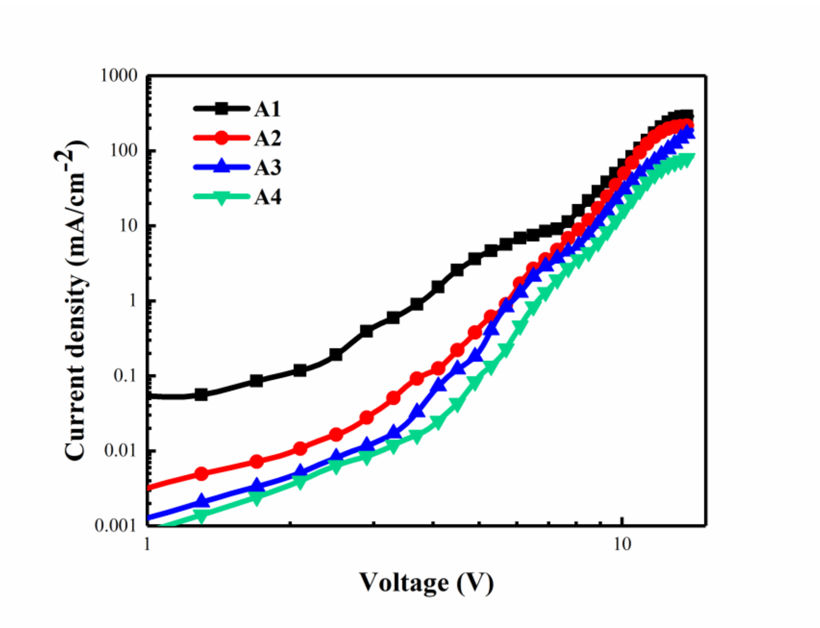

Figure S1. The double-logarithmic JV curves.

(a)

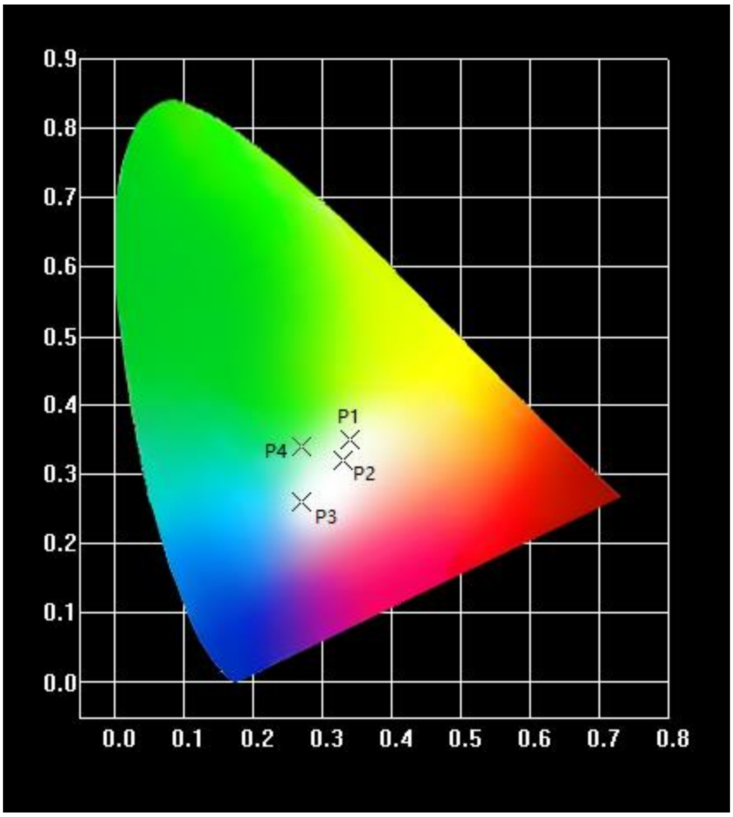

(b)

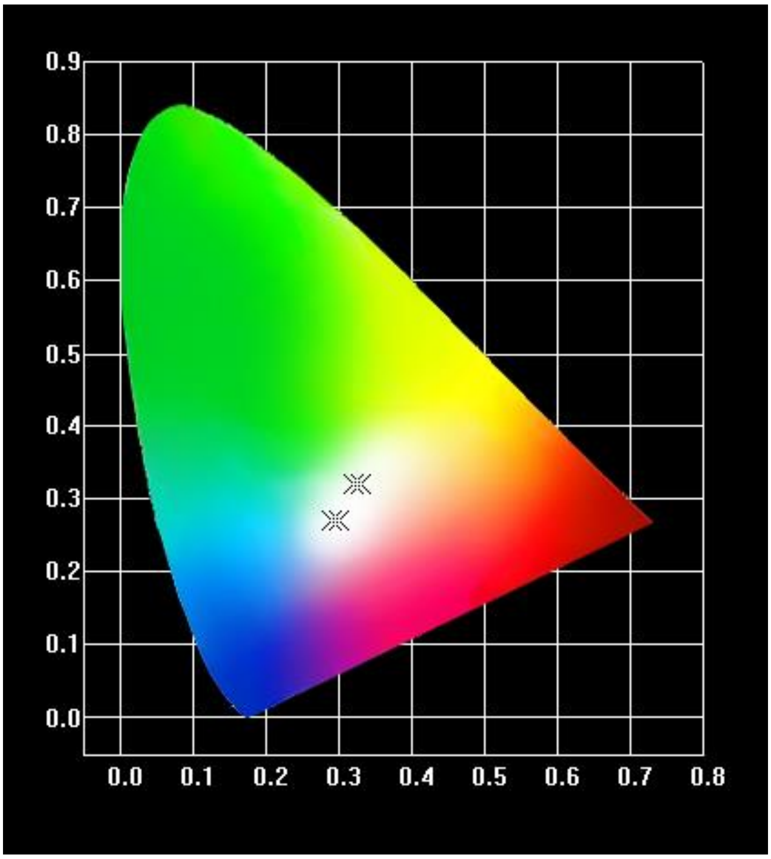

Figure S2. The 1931 CIE chromaticity diagram of emission of the WPLEDs; (**a**) the CIE coordinates of devices A1-A4 at a voltage of 10 V; (**b**) the CIE chromaticity diagram of device A2 at different voltages.

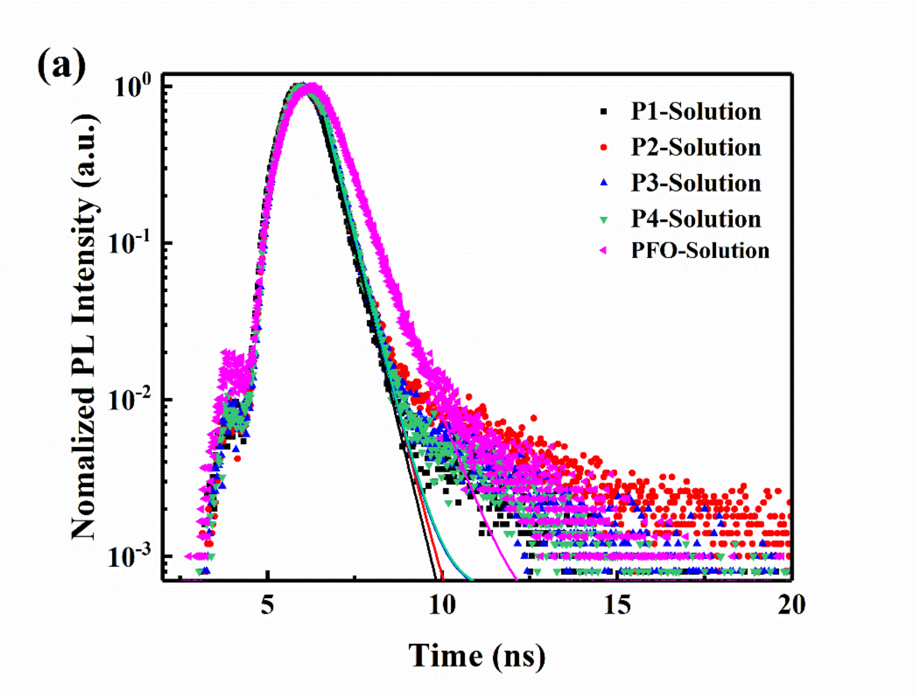

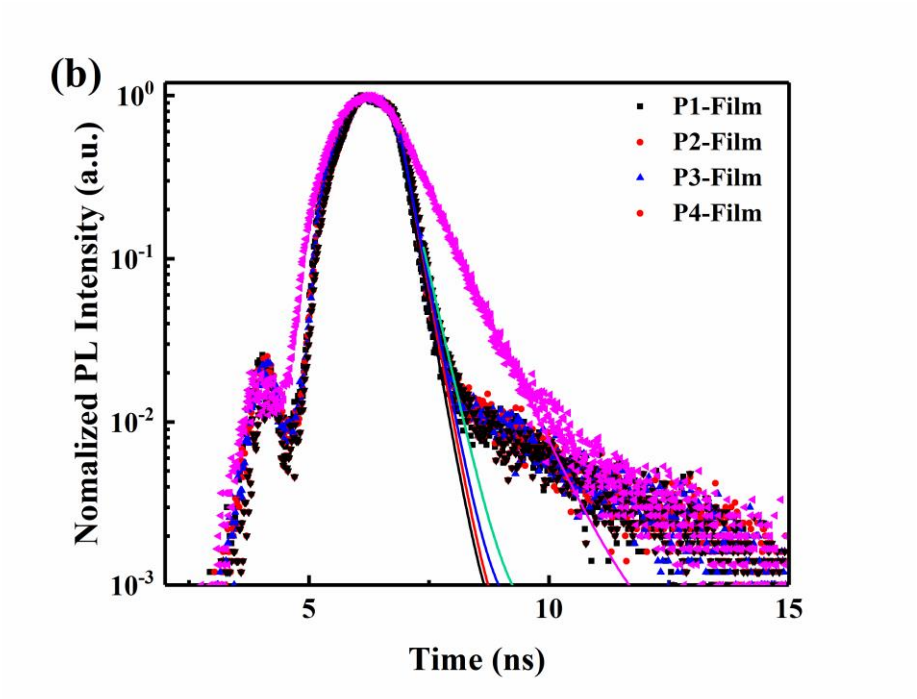

Figure S3. The fit curves of the time-resolved PL for copolymers P1-P4; (**a**) the white-light copolymer in solutions; (**b**) the white-light copolymer in films.
